# Effect of day 3 embryo morphometrics and morphokinetics on survival and implantation after slow freezing-thawing and after vitrification-warming: a retrospective cohort study

**DOI:** 10.1186/s12958-017-0299-5

**Published:** 2017-10-03

**Authors:** Elia Fernandez Gallardo, Carl Spiessens, Thomas D’Hooghe, Sophie Debrock

**Affiliations:** KU Leuven – University of Leuven, University Hospitals Leuven, Leuven University Fertility Center, Herestraat 49, B-3000 Leuven, Belgium

**Keywords:** Morphometrics, Morphokinetics, Human embryo, Vitrification, Slow freezing

## Abstract

**Background:**

Morphometric and morphokinetic evaluation of in vitro cultured human embryos allows evaluation without time restriction and reduces intra- and inter-observer variability. Even though these technologies have been reported to improve the quality of cleavage stage embryo evaluation during fresh culture, possible advantages in the evaluation of cryopreserved embryos have been scarcely explored. This study aims to compare morphometric and morphokinetic parameters between slow frozen and vitrified embryos and to determine their relationship to embryo survival and implantation rate (IR) after thawing/warming.

**Methods:**

During fresh culture, morphometric characteristics (Total Cell Volume (TCV), symmetry, fragmentation and number of blastomeres) were measured in 286 thawed/warmed embryos. Likewise, after thawing/warming, similar morphometric characteristics were measured in 135 survived embryos. Moreover, morphokinetic parameters (time to mitosis resumption and time to compaction) were measured in 90 embryos after thawing/warming. Then, using linear regression, we investigated the differences between vitrified and slow frozen embryos and the relation of the measured characteristics to embryo survival and IR. Statistical corrections were applied to account for data clustering and for multiple testing.

**Results:**

Vitrified embryos resume mitosis and start compaction significantly earlier than slow frozen embryos. Mitosis resumption rate was 82% for vitrified and 63% for slow frozen embryos and median time to mitosis resumption was 7.6 h and 13.1 h (*p* = 0.02), respectively. Compaction rate was 62% in vitrified and only 23% in slow frozen embryos. Median time to compaction was 18.1 h for vitrified embryos but, for slow frozen could not be computed since less than half of the slow frozen embryos reached compaction (*p* = 0.0001). Moreover, intact embryos resume mitosis significantly earlier than not intact ones regardless of the freezing method (rate: 79% vs. 66%, median time: 7.6 h vs 14.6 h, respectively, *p* = 0.03). Regarding morphometrics, slow frozen embryos showed lower TCV and higher blastomere symmetry after thawing than vitrified embryos despite having similar blastomere number. IR was related to blastomere number at cryopreservation in slow frozen embryos, but not in vitrified ones.

**Conclusions:**

Interestingly, vitrified/warmed embryos undergo mitosis resumption and compaction significantly earlier than slow frozen/thawed embryos. However, the clinical use of this morphokinetic parameters still remains to be investigated in larger studies.

**Trial registration:**

Retrospectively registered on December 15, 2015 NCT02639715.

## Background

Cryopreservation of supernumerary embryos generated after Assisted Reproductive Technology (ART) treatments allows an eventual frozen embryo transfer (FET) cycle and improves cumulative pregnancy rates. Supernumerary embryos are routinely cryopreserved either at cleavage stage (day 2 or 3) or at blastocyst stage (day 5 day 6). Since it is not yet clear which strategy is more beneficial for the FET outcome [1], we focused this study in our current clinical practice: cleavage stage embryo cryopreservation. The two available cryopreservation methods used for cleavage stage embryos at the moment are slow freezing and vitrification. Even though vitrification has proven to result in higher survival rates than slow freezing [2–4], slow frozen embryos can reach the same implantation rate per embryo transferred as vitrified embryos [5–7]. Regardless of the cryopreservation method, it is crucial to perform morphological evaluation of the embryos both, before cryopreservation and after thawing/warming to ensure sufficient embryo quality for transfer in the FET cycle.

Before cryopreservation, embryos are selected based on the morphological characteristics. As stated in the guidelines [8], only optimal cleavage stage embryos –i.e. 4-cell stage embryos at 44 ± 1 h post insemination, 8-cell stage embryos at 68 ± 1 h post insemination with <10% fragmentation, stage-specific cell size and no multinucleation [9]– should be cryopreserved. Nevertheless, in practice, most authors report less strict criteria, e.g. freezing embryos with up to 50% fragmentation [10, 11], with <8 and >8 blastomeres [6, 10, 12–21] and without taking cell size –i.e. symmetry– into consideration [6, 13, 15, 21–23], perhaps to avoid discarding embryos with acceptable implantation potential. The presence of asymmetrical blastomeres and the % fragmentation in fresh cleavage stage embryos have been negatively correlated with implantation rate (IR) [9] and positively correlated with the incidence of aneuploidy [24–27]. However, using a computer based morphometric analysis –that calculates both embryo symmetry and fragmentation based on individual blastomere volume and total cell volume (TCV) [28] –we did not observe any effect of neither symmetry (>50%) nor fragmentation (<40%) on IR in FET [29]. Yet, due to the fact that cryopreservation media cause exchange of water and medium through the cell membrane [8, 30] and because of the different concentration of cryoprotectants present in slow freezing and vitrification media [30], we hypothesize that TCV might influence differently the survival of slow frozen-thawed embryos than of vitrified-warmed embryos. Using morphometric evaluation we have previously shown that, indeed, vitrification can induce both an increase or decrease of TCV after warming, [29] and in fresh cycles, TCV has been associated with IR [28]. Currently, the effect of morphometric parameters on survival and IR after cryopreservation is still unexplored.

After thawing or warming, embryos are also morphologically evaluated. Two characteristics are routinely used: 1) the number of blastomeres survived and 2) the presence of mitosis resumption after overnight culture [8]. The IR is positively correlated with the number of blastomeres survived [21, 29, 31], even though there may be no difference in IR once at least 75% of blastomeres have survived [22, 23]. At the same time, the IR is positively correlated with the presence of mitosis resumption [21, 32, 33] even if the embryo has degenerated blastomeres after thawing or warming [6, 22]. In addition, we have previously observed that if compaction occurs during 20-24 h of overnight culture, there is no detrimental effect of the number of blastomeres degenerated after vitrification-warming on IR [29]. Despite the fact that the correlation between the number of blastomeres survived and the presence of mitosis resumption with IR in FET cycles has been extensively studied, the specific timing of mitosis resumption and compaction in cleavage stage embryos after thawing or warming has never been described and, more importantly, has never been related to IR in FET cycles. In concordance with the aforementioned hypothesis, the different composition and concentration of cryoprotectants in slow freezing compared to vitrification media [8, 30] might aswell have a different effect on the cell cycle recovery. Thus, we hypothesize that the time to mitosis resumption and time to compaction depends on the freezing method, and that this time interval may be shorter for embryos whose implantation leads to a pregnancy. Besides, the reported embryo TCV change caused by vitrification media [29] raises the question of whether slow freezing media also causes TCV change and, if contrarily to vitrification [29], has an effect on IR.

Importantly, morphometric and morphokinetic evaluation of cleavage stage embryos present several advantages compared to standard manual evaluation. As well as being non-invasive methods, they allow evaluation without time restriction [34] and reduce intra- and inter-observer variability [35, 36]. The benefits of applying these available technologies to cryopreserved embryos is scarcely explored and might help to improve the outcome of FET cycles by adapting the quality evaluation criteria of frozen-thawed/vitrified-warmed embryos.

The aims of this study are first, to describe and compare morphometric (TCV, symmetry and fragmentation) and morphokinetic (time to mitosis and time to compaction) parameters between slow frozen-thawed and vitrified-warmed cleavage stage embryos, and second, to determine their effect in embryo survival and IR after thawing/warming.

## Methods

### Patient and embryo selection

In this study, we used two different datasets: one set to measure morphometric parameters and the other set to measure morphokinetic parameters (Table 1).

**Table 1:** Number of embryos and characteristics in the databases used in this study. Emrbyos in dataset 1 were used for measurement of morphometric characteristics both during fresh culture and after thawing/warming. Embryos in dataset 2 were used for measurement of morphokinetic parameters after thawing/warming

	Slow frozen N (%)	Vitrified N (%)	Total N (%)
Embyos in dataset 1			
Fresh			
Total	159	127	286
Survived (Survived / Total)	79 (49.7)	112 (88.2)	191 (66.8)
Not recovered (Not recovered / Total)	8 (5.0)	1 (0.8)	9 (3.1)
Compacted at freezing (Compacted at freezing / Survived + Not survived)	14 (9.3)	8 (6.3)	22 (7.9)
Transferred (Transferred / Survived)	76 (96.2)	108 (96.4)	184 (96.3)
Implanted (Implanted/ Transferred)	13 (17.1)	20 (18.5)	33 (17.9)
Transferred in SET or DET with 0% or 100% implantation (Transferred in SET or DET with 0% or 100% implantation / Transferred)	71 (93.4)	89 (82.4)	160 (86.9)
Implanted^a^ (Implanted / Transferred in SET or DET with 0% or 100% implantation)	8 (11.3)	10 (11.2)	18 (11.2)
After thawing/warming			
Total^b^ (Total evaluated / Survived)	52 (65.8)	83 (74.1)	135 (70.7)
Compacted after thawing or warming (Compacted after thawing or warming / Total evaluated)	0 (0.0)	3 (3.6)	3 (2.2)
Compacted after overnight culture (Compacted after overnight culture / Total evaluated)	14 (26.9)	50 (60.2)	64 (47.4)
Transferred in SET or DET with 0% or 100% implantation (Transferred in SET or DET with 0% or 100% implantation among total evaluated / Total evaluated)	48 (92.3)	65 (78.3)	113 (83.7)
Implanted^c^ (Implanted/ Transferred in SET or DET with 0% or 100% implantation)	6 (12.5)	9 (13.8)	15 (13.3)
Embryos in dataset 2			
After thawing/warming			
Total	35	55	90
Intact (Intact/Total)	18 (51.4)	40 (72.7)	58 (64.4)
Resumed mitosis (Resumed mitosis/Total)	22 (62.8)	45 (81.8)	67 (74.4)
Transferred in SET or DET with 0% or 100% implantation (Transferred in SET or DET with 0% or 100% implantation / Total)	35 (100)	47 (85.4)	82 (91.1)
Implanted (Implanted / Transferred in SET or DET with 0% or 100% implantation)	4 (11.4)	10 (18.2)	14 (17.1)

^a^N implanted embryos among embryos transferred in SET or DET with 0% or 100% implantation

^b^Only survived embryos from which multilevel images were available after thawing or warming could be analyzed for morphometrics after thawing or warming

^c^N implanted embryos among evaluated embryos after thawing/warming transferred in SET or DET with 0% or 100% implantation

The first dataset (dataset 1) included 286 thawed/warmed embryos belonging to 109 patients who participated in a randomized control trial (RCT) comparing slow freezing versus vitrification with respect to live birth per embryo thawed/warmed [16]. Thus, morphometric parameters were measured from a total of 159 slow frozen embryos and 127 vitrified embryos (Table 1). The embryos were thawed/warmed between April 2012 and July 2014 in 163 FET cycles. Patient characteristics related to these embryos are described in Debrock et al., 2015 [16]. Briefly, only patients undergoing their first in vitro fertilization (IVF) cycle and younger than 40 years old were included. Cycles with PGD, oocyte reception or frozen/thawed oocytes were excluded.

The second dataset (dataset 2) included 90 thawed and survived embryos (Table 1) belonging to 70 patients who were treated during 77 FET cycles between July 2013 and August 2014, and who did not participate in the RCT mentioned above [16]. Thus, morphokinetic parameters were measured in a total of 35 slow frozen embryos and 55 vitrified embryos. Since in our lab the time lapse culture of thawed/warmed embryo is not incorporated in routine practice, and since our time lapse incubator (TLI) only provides place for two dishes (i.e. only the embryos from two patients could be recorded at the same time) we had to prospectively select which thawed/warmed embryos to culture in TLI. For that, we firstly excluded patients older than 40 years old, cycles with PGD, oocyte reception or frozen oocytes. Secondly, in case more than two patients were eligible for the study on the same day, we selected for TLI culture the thawed/warmed embryos of the two patients that had undergone the lowest number of fresh cycles previous to the current FET cycle.

All procedures were performed according to the Helsinki declaration on Human Experimentation. This study is in the frame of an approved project by the Commission for Medical Ethics of the University Hospital Leuven (approval reference number S55685) and the registered clinical trial (NCT02639715).

### Fresh cycle

In fresh cycles, ovarian stimulation and luteal supplementation were performed as described by Debrock et al., 2010 [37]. Oocyte retrieval (OR), insemination and embryo culture were carried out as in Debrock et al., 2015 [16]. Embryos were evaluated for fertilization on day 1 after OR (16-20 h after insemination/injection), and for quality on day 2 (41-44 h after insemination/injection) and day 3 (66-71 h after insemination/injection). Embryo quality was assessed using the manual scoring system of the Leuven University Fertility Centre which is based on the visual evaluation of the number and size of blastomeres and the degree of fragmentation [34]. At each evaluation moment, multilevel images of each embryo composed of 40 different focal planes were taken using Fertimorph Software (CellCura Software Solutions Copenhagen, Denmark) in order to perform image analysis retrospectively. On day 3, one or two embryos were chosen for transfer –according to the Belgian law [38]– based on the manual scoring system. Supernumerary embryos were cryopreserved on day 3 after OR if the embryos had at least 6 blastomeres, contained ≤25% fragmentation and had symmetrical to slight asymmetrical blastomeres (<50% size difference).

### Embryo cryopreservation

Two commercially available media were used: EmbryoStore Freeze (Gynemed, Lensahn, Germany) –with 1,2-propanediol and 0.1 M Sucrose as cryoprotectant – for slow freezing, and Vit Kit®–Freeze (Irvine Scientific, Newtownmountkennedy, Ireland) – with dimethylsulphoxide (DMSO)-ethylene glycol (EG)-sucrose as cryoprotectant –for vitrification. Both cryopreservation methods were performed following manufacturer’s protocol as described previously [16]. Slow frozen embryos were loaded into high security straws, introduced in a programmable freezer until −150 °C were reached. Then, they were plunged into liquid nitrogen. Vitrified embryos were loaded into CBS-VIT-High Security straws (CBS, Cryo Bio System, L’Aigle, France) and plunged directly into liquid nitrogen. Embryos were further stored in vapour phase nitrogen container.

### FET cycles, embryo evaluation and cryopreservation

Thawed/warmed embryos were transferred in natural cycles, stimulated cycles (gonadotrophin or clomiphene citrate) or hormonal replacement cycles as foresaid [39]. Straws were thawed/warmed following manufacturer’s protocol –EmbryoStore Thaw (Gynemed, Lensahn, Germany) and Vit Kit®-Thaw (Irvine Scientific, Newtownmountkennedy, Ireland) for slow frozen and for vitrified embryos respectively– until the number of survived embryos was equal to the number of requested embryos for transfer. A maximum of two embryos were replaced as determined by Belgian law [40].

After thawing/warming, embryos were cultured overnight in GM501 medium (Gynemed, Lensahn, Germany) under mineral oil (Gynemed) at 37 °C, pH 7.25–7.35 in a standard incubator (Sanyo MCO-20AIC, Osaka, Japan) or in a TLI (ASTEC Penguin incubator, Fukuoka, Japan). Embryo quality was assessed immediately after thawing/warming to evaluate survival, and after 22-24 h overnight culture to evaluate mitosis resumption. Embryos were considered survived if they had ≥50% of cells intact immediately after thawing. Embryos were considered intact if 100% of the blastomeres had survived. Mitosis resumption was defined as an increase of the number of blastomeres of the survived embryos after 20-24 h of overnight culture. No selection was performed in the thawed/warmed embryos for transfer. Thus, all embryos with or without mitosis resumption were transferred.

### Computer based morphometric analysis of embryos before and after cryopreservation and after overnight culture

Multilevel images that were taken before cryopreservation (on day 1 and day 3) and after thawing/warming (immediately after thawing/warming and again after overnight culture) were analysed using Fertimorph Software (CellCura Software Solutions Copenhagen, Denmark). This software calculates the TCV of the embryo based on the manual drawing of the two diameters of every blastomere [34]. Embryos with compaction could not be measured for morphometrics and resulted in missing values. In the measured embryos, the criteria to differentiate a blastomere and a fragment were based on the findings by Hnida et al. [41] and Johansson et al. [42]. A blastomere should be ≥40 μm on day 3 when the embryo has ≤8 blastomeres. When the embryo had >8 blastomeres, the minimum diameter for a blastomere was established as 35 μm.

Afterwards, TCV was used to calculate % of fragmentation and % blastomere symmetry as described in detail previously by Fernandez Gallardo et al., 2016 [29]. In summary, fragmentation was defined as the remaining volume (%) after substracting TCV_Day3_ to TCV_Day1_, thus, a bigger difference between TCV_Day1_ and TCV_Day3_ of the same embryo, results in higher % fragmentation. At the same time, symmetry was defined as the TCV similarity (%) between the biggest and the smallest blastomere of the embryo, thus, the higher the % of symmetry, the more similar is the size of the blastomeres.

### Morphokinetic analysis of thawed/warmed embryos during overnight culture

After thawing/warming, selected embryos were cultured overnight in the TLI for a minimum of 19 h and a maximum of 26 h. The time-lapse monitoring system used was ASTEC Penguin incubator (ASTEC Co., Fukuoka, Japan). The imaging system has a red LED as light source (620-630 nm wavelength) and consists in a CCM sensor camera unit connected to 10X phase contrast objective. The camera has a resolution of 1.4 megapixels and the size of the observed area is 640 μm × 480 μm. Every 5 min, images of each embryo were taken at 11 different focal planes separated by 10 μm. The time of illumination of each embryo at each time point was 467 msec. Images were visualized with an in-house built software that allowed to scroll the images through focal plane and through time point as well as to mark events in time and extract the data in timestamp format (i.e. date, hour and minute). This enabled the recording of two parameters for each embryo: time to mitosis resumption, which was defined as the time between the time of thawing and the time when the first cell cleavage was finished, and time to compaction, which was defined as the time between the time of thawing and the time when the membrane fusion of the first two (or more) blastomeres in the thawed/warmed embryo could be visualized.

### Study design and statistical analysis

#### Morphometrics

During fresh culture, morphometric characteristics (TCV, symmetry, fragmentation and number of blastomeres) of 286 thawed/warmed embryos were measured. Then, using linear regression, survived vs not survived embryos and implanted vs not implanted embryos were compared for each of the morphometric characteristics measured during fresh culture. Likewise, after thawing/warming, morphometric characteristics (TCV, symmetry and number of blastomeres) of 135 survived embryos were measured. Then, vitrified vs slow frozen embryos and implanted vs not implanted embryos were compared using linear regression for each of the morphometric characteristics measured after thawing/warming. To compare implanted vs not implanted embryos, only those transferred in single embryo transfers (SET) and double embryo transfer (DET) with either 0% or 100% implantation were included (*n* = 160 before freezing and *n* = 113 after thawing/warming) (Table 1). Since the dataset contains multiple embryos for the same patient, a random intercept was used for each subject to account for data clustering. Before interpreting *P*-values (α = 0.05) Bonferroni-Holm method was used per freezing method to correct for multiple testing. All statistical tests were performed using SAS software.

#### Morphokinetics

Morphokinetic characteristics after thawing/warming (time to mitosis and time to compaction) from 90 embryos were described using reverse Kaplan-Meier curves and were compared between slow frozen and vitrified embryos, between intact and not intact embryos and between implanted and not implanted embryos. Only embryos transferred in SET or in DET with 0% or 100% implantation were used to compare implanted vs not implanted embryos (Table 1). Groups were compared using clustered logrank test [43]. Since the dataset contains multiple embryos from the same patient, hazard ratios (HR) and 95% confidence intervals (CI) were based on simple random effects Cox proportional hazards regression models, modelling mother as random effect to account for data clustering. All statistical tests were performed using SAS software.

## Results

### Morphometric parameters during fresh culture of cryopreserved embryos

#### Survived vs not survived

In total, 159 slow frozen embryos and 127 vitrified embryos were thawed/warmed with a survival rate of 49.7% and 88.2%, respectively. Embryos that were not recovered after thawing/warming (8/159 and 1/127 respectively) were excluded from the analysis. Moreover, morphometric parameters from embryos that were compacted at freezing could not be measured (14/151 slow frozen and 8/126 vitrified embryos). Thus, in this analysis, 137 slow frozen (71 survived and 66 not survived) and 118 vitrified embryos (104 survived and 14 not survived) were included (Table 1). Morphometric parameters before freezing are described for survived and not survived embryos in Table 2. In slow frozen embryos, logistic regression shows no differences between survived and not survived embryos regarding any of the measured parameters during fresh culture (Table 2). In vitrified embryos, survived embryos had larger TCV (95% CI = 7159 μm^3^ to 112,858 μm^3^, *p* = 0.026), less % fragmentation (95% CI = −11.3% to 0.20%, *p* = 0.058) and less % blastomere symmetry (95% CI = −9.72% to −0.15%, *p* = 0.04) during fresh culture compared to not survived embryos, but these differences lost statistical significance after Bonferroni-Holm correction.

**Table 2:** Morphometric parameters during fresh culture before slow freezing or vitrification for survived and not survived embryos in dataset 1. Differences between survived and not survived embryos were calculated for each parameter using linear regression (95% CI and *p* values)

Freezing method	*Parameter*	Survived Mean ± SD	Not survived Mean ± SD	Coefficient survived vs not survived	95% CI	P
Slow freezing	*n*	71^b^	66^c^			
	*TCV, μm* ^*3*^	715,113 ± 86,190	719,250 ± 98,140	−1309	−30,401 to 27,783	0.9289
	*N blastomeres*	8.2 ± 1.9	8.1 ± 1.3	0.02	−0.55 to 0.59	0.9371
	*% Fragmentation* ^*a*^	13.7 ± 10.2	15.0 ± 9.6	−0.88	−4.25 to 2.49	0.6040
	*% Symmetry*	73.5 ± 8.2	71.1 ± 9.2	2.32	−0.63 to 5.28	0.1218
Vitrification	*n*	104^d^	14			
	*TCV, μm* ^*3*^	727,513 ± 101,655	688,627 ± 112,499	60,008	7159 to 112,858	0.0267
	*N blastomeres*	8.2 ± 1.8	8.9 ± 2.4	−0.19	−1.24 to 0.86	0.7164
	*% Fragmentation*	14.0 ± 10.0	19.3 ± 10.8	−5.55	−11.3 to 0.20	0.0584
	*% Symmetry*	72.4 ± 8.6	76.8 ± 6.8	−4.93	−9.72 to −0.15	0.0435

Random intercept correction was used to account for clustering

Bonferroni-Holm method was used per freezing method to correct for multiple testing

^a^1 embryo excluded because ≤ − 20% fragmentation

No morphometrics could be analyzed in compacted embryos

^b^8/79 embryos were compacted at evaluation

^c^6/72 embryos were compacted at evaluation

^d^8/112 embryos were compacted at evaluation

#### Implanted vs not implanted

A total of 184/286 survived embryos were transferred (76 slow frozen and 108 vitrified) with an overall implantation rate per embryo transferred of 17.1% for slow freezing and 18.5% for vitrification. Only embryos transferred in SET or in DET with 0% or 100% implantation (*n* = 160; 71 slow frozen and 89 vitrified) were used for further analysis, with implantation rates of 8/71 (11.3%) and 10/89 (11.2%) for the slow frozen group and the vitrified group, respectively (Table 1). Morphometric parameters during fresh culture of implanted and not implanted embryos are described Table 3. Morphometrics could not be measured in compacted embryos (15/160). While implanted and not implanted embryos did not differ in any characteristic in the vitrification group, implanted embryos had significantly higher number of blastomeres at freezing than not implanted embryos in the slow frozen group (95% CI = 0.93 to 3.68, *p* = 0.002) (Table 3).

**Table 3:** Morphometric parameters during fresh culture before slow freezing or vitrification for implanted and not implanted embryos in dataset 1. Differences between implanted and not implanted embryos were calculated for each parameter using linear regression (95% CI and *p* values)

Freezing method	*Parameter*	Implanted Mean ± SD	Not implanted Mean ± SD	Coefficient implanted vs not implanted	95% CI	P
Slow freezing	*n*	8	55^a^			
	*TCV, μm* ^*3*^	771,018 ± 96,123	710,644 ± 83,177	54,663	−11,047 to 120,372	0.0992
	*N blastomeres*	9.6 ± 3.0	7.9 ± 1.5	2.31	0.93 to 3.68	0.0020*
	*% Fragmentation*	8.6 ± 7.1	14.1 ± 10.6	−5.31	−13.4 to 2.81	0.1906
	*% Symmetry*	73.1 ± 4.9	73.9 ± 8.2	−1.00	−7.19 to 5.19	0.7418
Vitrification	*n*	10	72^b^			
	*TCV, μm* ^*3*^	738,603 ± 94,544	718,841 ± 1,037,167	12,538	−55,655 to 80,730	0.7120
	*N blastomeres*	8.7 ± 2.8	8.1 ± 1.7	0.81	−0.47 to 2.10	0.2073
	*% Fragmentation*	13.8 ± 8.4	14.6 ± 10.3	−0.85	−7.76 to 6.06	0.8049
	*% Symmetry*	72.1 ± 9.7	72.2 ± 8.8	−0.29	−6.45 to 5.86	0.9234

Random intercept correction was used to account for clustering

*Statistically significant (at 5% alpha level) using the Bonferroni-Holm correction for multiple testing

No morphometrics could be analyzed in compacted embryos

^a^8/63 were compacted at evaluation

^b^7/79 were compacted at evaluation

### Morphometric parameters after thawing/warming of survived embryos

#### Slow frozen vs. vitrified

Morphometric parameters were evaluated in 52/79 survived embryos after slow freezing/thawing and in 83/112 survived embryos after vitrification/warming. Due to missing images after thawing/warming 27/79 embryos and 29/112 embryos were excluded, respectively. Morphometrics could not be measured in compacted embryos (*n* = 3 after thawing; *n* = 64 after overnight culture). Among the survived embryos with available images (52 slow frozen and 83 vitrified), the intact survival rate was 42.3% after slow freezing and 74.7% after vitrification (Table 1). Table 4 shows the description of morphometric parameters after thawing and after overnight culture for both freezing methods. Linear regression showed that TCV decreases during the culture after thawing (95% CI = −102,621 μm^3^ to -27,468 μm^3^, *p* = 0.0008), while the number of blastomeres increases (95% CI = 0.89 to 2.26, p = <0.0001) however none of the *p* values was significant after correcting for multiple testing. Moreover, slow frozen embryos showed significantly lower TCV (95% CI = −193,476 μm^3^ to −92,686 μm^3^, *p* < 0.0001) and significantly higher blastomere symmetry (95% CI = 0.77% to 7.18%, *p* = 0.01) after thawing compared to vitrified embryos (Table 4). Nevertheless, number of blastomeres was comparable between the two groups (95% CI = −2.05 to 0.09, *p* = 0.07).

**Table 4:** Morphometric parameters after thawing for survived slow frozen and vitrified embryos in dataset 1. Differences between survived slow frozen and vitrified embryos, and between day of evaluation, were calculated for each parameter using linear regression (95% CI and p values)

	Day of measurement	Slow frozen Mean ± SD	Vitrified Mean ± SD	Coefficient (95%CI) Freezing method	*P* value Freezing method	Coefficient (95% CI) Day	P value Day
N embryos		52 ^a^	80 ^b, c^				
TCV (μm^3^)	At thaw/warming	550,798 ± 162,603	702,510 ± 12,793	−143,081 (193,476 to −92,686)	<0.0001*	−65,045 (−102,621 to −27,468)	0.0008
	After O/N culture	505,520 ± 144,231	607,813 ± 89,982				
Blastomeres (n)	At thaw/warming	7.1 ± 2.6	8.3 ± 2.3	−0.98 (−2.05 to 0.09)	0.0729	1.58 (0.89 to 2.26)	<0.0001
	After O/N culture	8.5 ± 3.4	9.6 ± 3.0				
Symmetry (%)	At thaw/warming	76.4 ± 8.7	72.4 ± 9.6	3.97 (0.77 to 7.18)	0.0155*	−6.46 (−8.82 to −4.09)	<0.0001
	After O/N culture	70.5 ± 8.9	66.4 ± 6.7				

*Statistically significant (at 5% alpha level) using the Bonferroni-Holm correction for multiple testing to test for any difference between slow frozen and vitrified embryos

Random intercept correction was used to account for clustering

^a^14/52 embryos were compacted after overnight culture

^b^3/83 embryos were compacted after thawing

^c^50/83 embryos were compacted after overnight culture

#### Implanted vs. not implanted

From the 135 embryos analysed for morphometric characteristics after thawing/warming (52 slow frozen and 83 vitrified) only 48 slow frozen and 65 vitrified were transferred in SET or DET with 0% or 100% implantation, with IRs of 6/48 (12.5%) and 9/65 (13.8%) in the slow frozen group and in the vitrification group, respectively (Table 1). Due to the high number of compacted embryos after overnight culture, whose morphometric parameters could not be measured, only measurements after thawing/warming are included in this analysis, but not measurements after overnight culture. Table 5 contains morphometric parameters after thawing/warming and linear regression results. Implanted and not implanted embryos did not show any significant difference regarding any of the measured parameters either after slow freezing or vitrification. In the slow frozen group, borderline *p*-values resulted from comparing TCV (95% CI = −271,131 μm^3^ to 1689 μm^3^, *p* = 0.05) and number of blastomeres (95% CI = −0.1 to 4.5, p = 0.05) of implanted and not implanted embryos.

**Table 5:** Morphometric parameters after thawing/warming for implanted and not implanted embryos in dataset 1. Differences between implanted and not implanted embryos were calculated for each parameter using linear regression (95% CI and p values)

Freezing method	*Parameter*	Implanted Mean ± SD	Not implanted Mean ± SD	Coefficient implanted vs not implanted	95% CI	P
	*n*	6	42			
Slow freezing	*TCV, μm* ^*3*^	666,658 ± 167,567	531,951 ± 143,637	134,721	−1689 to 271,131	0.0526
	*N blastomeres*	9.0 ± 2.5	6.8 ± 2.4	2.2	−0.1 to 4.5	0.0557
	*% Symmetry*	75.0 ± 7.2	77.1 ± 9.2	−1.9	−10.3 to 6.6	0.6465
	*n*	9	53^a^			
Vitrification	*TCV, μm* ^*3*^	729,666 ± 102,954	693,512 ± 137,507	24,599	−76,439 to 125,638	0.6219
	*N blastomeres*	9.4 ± 2.8	8.2 ± 2.4	1.2	−0.6 to 3.1	0.1883
	*% Symmetry*	67.4 ± 9.3	71.5 ± 9.6	−4.0	−10.9 to 2.8	0.2364

Random intercept correction was used to account for clustering

Bonferroni-Holm method was used per freezing method to correct for multiple testing

No morphometrics could be analyzed in compacted embryos

^a^3 embryos were compacted after thawing

### Morphokinetic characteristics after thawing/warming

**Fig. 1:**
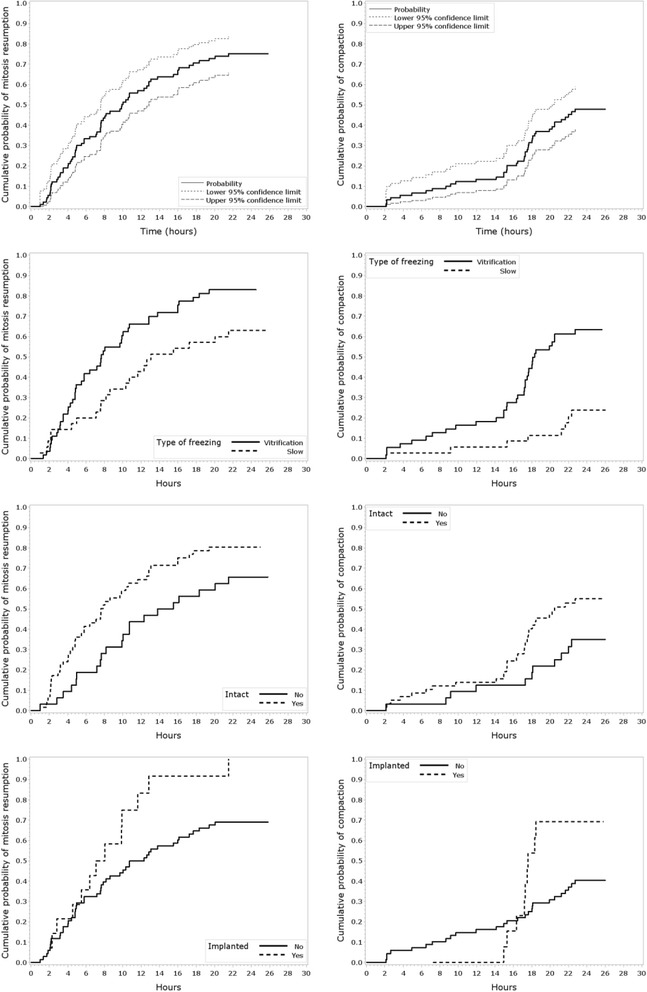
Reverse Kaplan-Meier curves for time to mitosis resumption and time to compaction after thawing/warming of human cryopreserved embryos in dataset 2. The groups of vitrified and slow frozen embryos, of intact and not intact embryos and of implanted and non-implanted embryos were compared for both morphokinetic characteristics. Clustered log rank test p-values indicated a significantly faster mitosis resumption (*p* = 0.0185) and faster compaction (p = 0.0001) in vitrified embryos compared to slow frozen, and significantly faster mitosis resumption in intact embryos compared to not intact (p = 0.03). All other comparissons showed no significant differences

Time to mitosis resumption and time to compaction was measured in 90 survived embryos after cryopreservation (dataset 2: 35 slow frozen and 55 vitrified). From those, 82 embryos were transferred in SET or DET with 0% or 100% implantation, and were used to compare implanted (*n* = 14) and not implanted embryos (*n* = 68) (Table 1). Reverse Kaplan-Meier curves show the proportion of embryos with mitosis resumption and with compaction along the time, separately for type of freezing, intact/not intact and implanted/not implanted (Fig. 1). The proportion of embryos with mitosis resumption and compaction, the median time to reach both characteristics and the logrank test HR (95% CI) and *P* values are collected in Table 6. Reverse Kaplan-Meier curves show that vitrified embryos resume mitosis (*p* = 0.02) and start compaction earlier (*p* = 0.0001) than slow frozen embryos. When comparing intact and not intact embryos reverse Kaplan-Meier curves, intact embryos showed a significantly earlier mitosis resumption i (*p* = 0.03) and a trend towards earlier compaction (*p* = 0.06). In contrast, implanted and not implanted embryos did not reach significant difference in neither the time to mitosis nor the time to compaction.

**Table 6:** Morphokinetic characteristics of human cryopreserved embryos after thawing/warming in dataset 2. *P*-values are based on a clustered logrank test. Hazard ratios and 95% CIs are based on simple random effects Cox proportional hazards regression models, modeling mother as random effect

	N cells at freezing mean±SD	Mitosis resumption N (%)	Hours to mitosis resumption Median	HR	95% CI	P	Compaction N (%)	Hours to Compaction Median	HR	95% CI	P
All embryos	8.1±1.5	67 (74)	9.9				42 (47)	NA			
Type of freezing											
Slow freezing	8.0±1.6	22 (63)	13.1	0.49	0.27 to 0.88	0.0185	8 (23)	NA	0.22	0.10 to 0.51	0.0001
Vitrification	8.1±1.5	45 (82)	7.6				34 (62)	18.1			
Intact											
No	8.1±1.7	21 (66)	14.6	1.86	1.04 to 3.31	0.0335	11 (34)	NA	2.28	1.03 to 5.05	0.0638
Yes	8.1±1.4	46 (79)	7.6				31 (53)	20.1			
Implanted											
No	8.2±1.7	47 (69)	11.5	nc	nc	0.2062	27 (40)	NA	nc	nc	0.1345
Yes	7.6±0.6	13 (93)	7.2				9 (64)	17.6			

*Nc* not computed because the model did not converge

*NA* median time cannot be computed as less than half of the embryos reached the outcome

## Discussion

Morphometric and morphokinetic evaluation of in vitro cultured human embryos allow evaluation without time restriction [34] and reduce intra- and inter-observer variability [35, 36]. Even though these technologies have been reported to improve cleavage stage embryo quality evaluation during fresh culture [34, 44], the advantages that they confer in the evaluation of cryopreserved embryos have been scarcely explored. For the first time, to the best of our knowledge, we investigate the differences between slow frozen and vitrified embryos with respect to TCV and symmetry –measured using computer assisted morphometrics after cryopreservation– as well as to time to mitosis resumption and time to compaction –observed during 19-26 h of overnight culture in TLI. In addition, we studied the effect of the aforementioned parameters with survival after thawing/warming and/or IR in FET cycles. We found that vitrified embryos did resume mitosis and start compaction significantly earlier than slow frozen embryos, and the same trend was observed in implanted embryos compared to not implanted embryos. In contrast to our hypothesis, among all the morphometric and morphokinetic parameters evaluated, only number of blastomeres at cryopreservation was found to be significantly related to IR in slow frozen embryos, and none of the other parameters studied were related to either survival or IR after thawing/warming.

Currently, the key performance indicators of cleavage stage embryo cryopreservation are survival rate, intact survival rate and mitosis resumption rate [8]. Survival and intact survival have different benchmark values for the two cryopreservation techniques, being 60% and 40% respectively for slow freezing and 85% and 70%, for vitrification [8]. In our study, survival and intact survival rates surpassed the benchmark level, both for slow freezing (49.7% and 42.3%) and for vitrification (88.2% and 74.7%). For mitosis resumption rate, as well as for IR, the guidelines establish a ≤ 10% of maximum decrease in relation to the comparable fresh embryo population. In our clinic, fresh transfers are performed on day 3, so there is no available day 3 - day 4 fresh embryo population to compare with. Nevertheless, our mitosis resumption rate (76.9% for slow freezing and 92.8% for vitrification) and our IR (17.1% for slow freezing and 18.5% for vitrification) are comparable to previous studies [6, 16, 19]. In summary, the cryopreserved embryo cohort presented in this study reaches the quality benchmarks established by experts.

Previous studies that report the positive association between presence of mitosis resumption after thawing/warming and IR [6, 21, 29, 33] are based on a single observation of the embryos. In the present study, by observing the embryo development under time lapse, we could describe the time of mitosis resumption as well as the time to compaction, delivering novel information about embryo development after thawing/warming. Timings of the first embryo cell divisions have been previously described only in fresh embryos e.g., the cleavage from 5 to 8 cells takes 40 ± 10 min, the interphase in 8 cells lasts for 23 ± 1 h and the cleavage from 8 to 16 cells takes 55 ± 15 min [45]. The fact that our cohort contains embryos at different cell stages – i.e. cleaving from 6 to 8 cells or from 8 to 16 cells – complicates the determination of an expected cleavage timing after thawing/warming based on the findings in fresh embryos. Nevertheless, the fact that slow frozen embryos have a lower mitosis resumption rate [6, 16, 19] supports our finding that slow frozen embryos take double the time as vitrified embryos to resume mitosis or to start compaction (Table 6). Interestingly, timings described in fresh embryos suggest that embryos frozen at the 8-cell stage might take longer time to resume mitosis compared to embryos in other cell stages. Future studies could investigate the importance of the cell stage at freezing related to the time to mitosis resumption. With time lapse, we could also observe that embryos with at least one cell damaged cleave significantly later than embryos that survive intact, regardless of the freezing method (Table 6). Even though these timings were significantly different between slow freezing and vitrification and between intact and not intact embryos, we could not confirm a significant effect of time to mitosis resumption and time to compaction on IR, perhaps due to the small embryo cohort remaining after excluding the embryos transferred in DET with 50% implantation (*n* = 82). It would be interesting to evaluate the clinical use of time to mitosis resumption in cryopreserved embryos with larger studies.

In contrast to our hypothesis, morphometric parameters of cleavage stage embryos both before cryopreservation and after thawing/warming, were neither related to survival nor to IR. We previously described, for a bigger cohort of vitrified embryos, that IR was not related to any of the morphometric parameters (symmetry, fragmentation and TCV) measured after warming [29]. We describe now that these measurements might neither improve the evaluation of slow frozen embryos nor the selection of embryos for cryopreservation. Interestingly, the only morphological parameter related to IR in the present embryo cohort was the number of blastomeres at cryopreservation, and only when using slow freezing method. This again confirms the widely described inferior intact survival rate of slow frozen embryos compared to vitrified ones [6, 16, 19]. In this regard, in case of using slow freezing, embryos should be frozen only if they have at least 8 cells whereas vitrification has proven to be successful with lower blastomeres number [29].

Despite the fact that we could not describe any new parameter to evaluate at the embryo selection for cryopreservation or for transfer after thawing/warming, we did reveal differences between slow frozen and vitrified embryos. After thawing, besides a significantly lower TCV – due to the lower intact survival rate of slow freezing –, slow frozen embryos had higher blastomere symmetry compared to vitrified embryos, which was not present before cryopreservation. In fact, the symmetry in embryos selected for cryopreservation is high (>70%) because the majority are in 8-cell stage. Eight cell embryos have the most symmetrical blastomeres, together with 2, and 4 cells [46]. Even though slow frozen/thawed embryos have a higher number of blastomeres degenerated, we expect that blastomeres maintain the size, and thus, remain symmetrical. Moreover, vitrified/warmed embryos have a higher mitosis resumption rate and compaction than slow frozen/thawed embryos [6], which means that they grow over the 8-cell stage, lowering, therefore, the symmetry [29, 46].

## Conclusion

Our study confirms that intact survival and mitosis resumption remain the most clinically important parameters in the evaluation of implantation potential of cryopreserved embryos, especially after slow freezing. Interestingly, vitrified/warmed embryos undergo mitosis resumption and compaction significantly earlier than slow frozen/thawed embryos. However, the clinical use of this morphokinetic parameters still remains to be investigated in larger studies. Unfortunately, morphometric characteristics do not improve the embryo evaluation after cryopreservation with none of the freezing methods.

## References

[1] Glujovsky D, Farquhar C, Quinteiro Retamar AM, Alvarez Sedo CR, Blake D (2016). Cleavage stage versus blastocyst stage embryo transfer in assisted reproductive technology. Cochrane Database Syst Rev.

[2] Loutradi KE, Kolibianakis EM, Venetis CA, Papanikolaou EG, Pados G, Bontis I (2008). Cryopreservation of human embryos by vitrification or slow freezing: a systematic review and meta-analysis. Fertil Steril.

[3] Kolibianakis EM, Venetis CA, Tarlatzis BC (2009). Cryopreservation of human embryos by vitrification or slow freezing: which one is better?. Curr Opin Obstet Gynecol.

[4] AbdelHafez FF, Desai N, Abou-Setta AM, Falcone T, Goldfarb J (2010). Slow freezing, vitrification and ultra-rapid freezing of human embryos: a systematic review and meta-analysis. Reprod BioMed Online.

[5] Edgar DH, Gook DA (2012). A critical appraisal of cryopreservation (slow cooling versus vitrification) of human oocytes and embryos. Hum Reprod Update.

[6] Van Landuyt L, Van de Velde H, De Vos A, Haentjens P, Blockeel C, Tournaye H (2013). Influence of cell loss after vitrification or slow-freezing on further in vitro development and implantation of human Day 3 embryos. Hum Reprod.

[7] Zhu HY, Xue YM, Yang LY, Jiang LY, Ling C, Tong XM (2015). Slow freezing should not be totally substituted by vitrification when applied to day 3 embryo cryopreservation: an analysis of 5613 frozen cycles. J Assist Reprod Genet.

[8] Alpha Scientists In Reproductive Medicine (2012). The Alpha consensus meeting on cryopreservation key performance indicators and benchmarks: proceedings of an expert meeting. Reprod BioMed Online.

[9] Alpha Scientists in Reproductive Medicine and Eshre Special Interest Group of Embryology (2011). The Istanbul consensus workshop on embryo assessment: proceedings of an expert meeting. Hum Reprod.

[10] Xue Y, Tong X, Jiang L, Zhu H, Yang L, Zhang S (2014). Effect of vitrification versus slow freezing of human day 3 embryos on beta-hCG levels. J Assist Reprod Genet.

[11] Veleva Z, Orava M, Nuojua-Huttunen S, Tapanainen JS, Martikainen H (2013). Factors affecting the outcome of frozen-thawed embryo transfer. Hum Reprod.

[12] Balaban B, Urman B, Ata B, Isiklar A, Larman MG, Hamilton R (2008). A randomized controlled study of human Day 3 embryo cryopreservation by slow freezing or vitrification: vitrification is associated with higher survival, metabolism and blastocyst formation. Hum Reprod.

[13] Cercas R, Villas C, Pons I, Brana C, Fernandez-Shaw S (2012). Vitrification can modify embryo cleavage stage after warming. Should we change endometrial preparation?. J Assist Reprod Genet.

[14] Chi F, Luo C, Yin P, Hong L, Ruan J, Huang M (2015). Vitrification of day 3 cleavage-stage embryos yields better clinical outcome in comparison with vitrification of day 2 cleavage-stage embryos. Zygote.

[15] Cobo A, de los Santos MJ, Castello D, Gamiz P, Campos P, Remohi J (2012). Outcomes of vitrified early cleavage-stage and blastocyst-stage embryos in a cryopreservation program: evaluation of 3,150 warming cycles. Fertil Steril.

[16] Debrock S, Peeraer K, Fernandez Gallardo E, De Neubourg D, Spiessens C, D'Hooghe TM (2015). Vitrification of cleavage stage day 3 embryos results in higher live birth rates than conventional slow freezing: a RCT. Hum Reprod.

[17] Desai N, Blackmon H, Szeptycki J, Goldfarb J (2007). Cryoloop vitrification of human day 3 cleavage-stage embryos: post-vitrification development, pregnancy outcomes and live births. Reprod BioMed Online.

[18] El-Danasouri I, Selman H (2001). Successful pregnancies and deliveries after a simple vitrification protocol for day 3 human embryos. Fertil Steril.

[19] Fasano G, Fontenelle N, Vannin AS, Biramane J, Devreker F, Englert Y (2014). A randomized controlled trial comparing two vitrification methods versus slow-freezing for cryopreservation of human cleavage stage embryos. J Assist Reprod Genet.

[20] Wilding MG, Capobianco C, Montanaro N, Kabili G, Di Matteo L, Fusco E (2010). Human cleavage-stage embryo vitrification is comparable to slow-rate cryopreservation in cycles of assisted reproduction. J Assist Reprod Genet.

[21] Sole M, Santalo J, Rodriguez I, Boada M, Coroleu B, Barri PN (2011). Correlation between embryological factors and pregnancy rate: development of an embryo score in a cryopreservation programme. J Assist Reprod Genet.

[22] Tang R, Catt J, Howlett D (2006). Towards defining parameters for a successful single embryo transfer in frozen cycles. Hum Reprod.

[23] Zheng X, Liu P, Chen G, Qiao J, Wu Y, Fan M (2008). Viability of frozen-thawed human embryos with one-two blastomeres lysis. J Assist Reprod Genet.

[24] Ziebe S, Lundin K, Loft A, Bergh C, Nyboe Andersen A, Selleskog U (2003). FISH analysis for chromosomes 13, 16, 18, 21, 22, X and Y in all blastomeres of IVF pre-embryos from 144 randomly selected donated human oocytes and impact on pre-embryo morphology. Hum Reprod.

[25] Munne S (2006). Chromosome abnormalities and their relationship to morphology and development of human embryos. Reprod BioMed Online.

[26] Giorgetti C, Terriou P, Auquier P, Hans E, Spach JL, Salzmann J (1995). Embryo score to predict implantation after in-vitro fertilization: based on 957 single embryo transfers. Hum Reprod.

[27] Hardarson T, Hanson C, Sjogren A, Lundin K (2001). Human embryos with unevenly sized blastomeres have lower pregnancy and implantation rates: indications for aneuploidy and multinucleation. Hum Reprod.

[28] Paternot G, Debrock S, De Neubourg D, D'Hooghe TM, Spiessens C (2013). Semi-automated morphometric analysis of human embryos can reveal correlations between total embryo volume and clinical pregnancy. Hum Reprod.

[29] Fernandez Gallardo E, Spiessens C, D'Hooghe T, Debrock S (2016). Effect of embryo morphology and morphometrics on implantation of vitrified day 3 embryos after warming: a retrospective cohort study. Reprod Biol Endocrinol.

[30] Wong KM, Mastenbroek S, Repping S (2014). Cryopreservation of human embryos and its contribution to in vitro fertilization success rates. Fertil Steril.

[31] Van den Abbeel E, Camus M, Van Waesberghe L, Devroey P, Van Steirteghem AC (1997). Viability of partially damaged human embryos after cryopreservation. Hum Reprod.

[32] Van der Elst J, Van den Abbeel E, Vitrier S, Camus M, Devroey P, Van Steirteghem AC (1997). Selective transfer of cryopreserved human embryos with further cleavage after thawing increases delivery and implantation rates. Hum Reprod.

[33] Joshi BV, Banker MR, Patel PM, Shah PB (2010). Transfer of human frozen-thawed embryos with further cleavage during culture increases pregnancy rates. J Hum Reprod Sci.

[34] Paternot G, Debrock S, D'Hooghe T, Spiessens C (2011). Computer-assisted embryo selection: a benefit in the evaluation of embryo quality?. Reprod BioMed Online.

[35] Paternot G, Devroe J, Debrock S, D'Hooghe TM, Spiessens C (2009). Intra- and inter-observer analysis in the morphological assessment of early-stage embryos. Reprod Biol Endocrinol.

[36] Sundvall L, Ingerslev HJ, Breth Knudsen U, Kirkegaard K (2013). Inter- and intra-observer variability of time-lapse annotations. Hum Reprod.

[37] Debrock S, Melotte C, Spiessens C, Peeraer K, Vanneste E, Meeuwis L (2010). Preimplantation genetic screening for aneuploidy of embryos after in vitro fertilization in women aged at least 35 years: a prospective randomized trial. Fertil Steril.

[38] De Neubourg D, Bogaerts K, Wyns C, Albert A, Camus M, Candeur M (2013). The history of Belgian assisted reproduction technology cycle registration and control: a case study in reducing the incidence of multiple pregnancy. Hum Reprod.

[39] Debrock S, Peeraer K, Spiessens C, Willemen D, De Loecker P, D'Hooghe TM (2011). The effect of modified quarter laser-assisted zona thinning on the implantation rate per embryo in frozen/vitrified-thawed/warmed embryo transfer cycles: a prospective randomized controlled trial. Hum Reprod.

[40] Wet van 6/07/2007 betreffende de medisch begeleide voortplanting en de bestemming van de overtallige embryo’s en de gameten, art 9 (June 2007).

[41] Hnida C, Agerholm I, Ziebe S (2005). Traditional detection versus computer-controlled multilevel analysis of nuclear structures from donated human embryos. Hum Reprod.

[42] Johansson M, Hardarson T, Lundin K (2003). There is a cutoff limit in diameter between a blastomere and a small anucleate fragment. J Assist Reprod Genet.

[43] Stedman MR, Gagnon DR, Lew RA, Jung SH, Losina E, Brookhart MA (2011). A SAS macro for a clustered logrank test. Comput Methods Prog Biomed.

[44] Rubio I, Galan A, Larreategui Z, Ayerdi F, Bellver J, Herrero J (2014). Clinical validation of embryo culture and selection by morphokinetic analysis: a randomized, controlled trial of the EmbryoScope. Fertil Steril.

[45] Kaser DJ, Racowsky C (2014). Clinical outcomes following selection of human preimplantation embryos with time-lapse monitoring: a systematic review. Hum Reprod Update.

[46] Goyanes V, Cupeiro A, Campos A, Lage B, Garcia Alonso L, Cornelissen G (1995). Circadian rhythm of sister chromatid exchanges in human chromosomes. In Vivo.

